# An autopsy report of three kindred in a Gerstmann–Sträussler–Scheinker disease P105L family with a special reference to prion protein, tau, and beta‐amyloid

**DOI:** 10.1002/brb3.1117

**Published:** 2018-09-21

**Authors:** Keisuke Ishizawa, Takashi Mitsufuji, Kei Shioda, Atsushi Kobayashi, Takashi Komori, Yoshihiko Nakazato, Tetsuyuki Kitamoto, Nobuo Araki, Toshimasa Yamamoto, Atsushi Sasaki

**Affiliations:** ^1^ Department of Neurology Saitama Medical University Saitama Japan; ^2^ Department of Pathology Saitama Medical University Saitama Japan; ^3^ Hokkaido University Graduate School of Veterinary Medicine Hokkaido Japan; ^4^ Department of Pathology Tokyo Metropolitan Neurological Hospital Tokyo Japan; ^5^ Division of CJD Science and Technology, Department of Prion Research, Center for Translational and Advanced Animal Research on Human Diseases Tohoku University Graduate School of Medicine Miyagi Japan

**Keywords:** autopsy, beta‐amyloid, Gerstmann–Sträussler–Scheinker disease P105L, prion protein, spastic paraparesis, tau

## Abstract

**Introduction:**

Gerstmann–Sträussler–Scheinker disease P105L (GSS105) is a rare variant of GSS caused by a point mutation of the prion protein (PrP) gene at codon 105 (proline to leucine substitution). It is clinically characterized by spastic paraparesis and dementia and histopathologically defined by PrP‐plaques in the brain. This report describes a clinicopathological analysis of three autopsied kindred from a Japanese GSS105 family, plus a topological analysis of PrP, hyperphosphorylated tau (p‐tau), and beta‐amyloid (Aβ).

**Methods:**

Using paraffin‐embedded sections, we applied histology and single‐ and multiple‐labeling immunohistochemistry for PrP, p‐tau, and Aβ to the three cases. Comparative semi‐quantitative analyses of tissue injuries and PrP‐plaques were also employed.

**Results:**

Case 1 (45 years old (yo)) and Case 2 (56 yo) are sisters, and Case 3 (49 yo) is the son of Case 2. Case 1 and Case 2 presented with spastic paraparesis followed by dementia, whereas Case 3 presented, not with spastic paraparesis, but with psychiatric symptoms. In Case 1 and Case 2, the brain showed tissue injuries with many PrP‐plaques in the cerebral cortices, and the pyramidal tract showed myelin loss/pallor. In Case 3, the brain was least degenerated with a number of PrP‐plaques; however, the pyramidal tract remained intact. In addition, p‐tau was deposited in all cases, where p‐tau was present in or around PrP‐plaques. By double‐labeling immunohistochemistry, the colocalization of p‐tau with PrP‐plaques was confirmed. Moreover in Case 2, Aβ was deposited in the cerebral cortices. Interestingly, not only p‐tau but also Aβ was colocalized with PrP‐plaques. In all cases, both three repeat tau and four repeat tau were associated with PrP‐plaques.

**Conclusions:**

The clinicopathological diversity of GSS105, which is possible even in the same family, was ascertained. Not only p‐tau but also Aβ could be induced by PrP (“secondary degeneration”), facilitating the kaleidoscopic symptoms of GSS.

## 1 INTRODUCTION

Gerstmann–Sträussler–Scheinker disease (GSS) is an autosomal dominant neurodegenerative disorder caused by prion protein (PrP) gene (*PRNP*) mutations on chromosome 20 (Ghetti, Tagliavini, Kovacs, & Piccardo, 2011). GSS is clinically characterized by a constellation of signs and symptoms, and the cardinal neuropathological feature of GSS is the formation of amyloid plaques composed of PrP (PrP‐plaques) that are most abundant in the cerebral cortex, basal ganglia, and cerebellar cortex (Ghetti et al., 2011). GSS P105L (GSS105), a rare variant of GSS caused by a point mutation of *PRNP* at codon 105 (proline to leucine substitution), was first reported in Japan (Kitamoto, Amano, et al., 1993; Yamada et al., 1993) and is clinically characterized by gait disturbance (spastic paraparesis), dementia, or psychiatric disorders (Amano et al., 1992; Isshiki, Minagawa, & Yamauchi, 1994; Itoh et al., 1994; Kitamoto, Amano, et al., 1993; Kubo, Nishimura, Shikata, Kokubun, & Takasu, 1995; Nakazato, Ohno, Negishi, Hamaguchi, & Arai, 1991; Yamada et al., 1993).

More than 20 years ago, two members of a Japanese GSS105 family were reported as separate case reports in Japanese (Isshiki et al., 1994; Nakazato et al., 1991). Both of these cases presented with spastic paraparesis and later with dementia and so on, and were subjected to autopsy. Neuropathological examination disclosed numerous PrP‐plaques in the cerebrum and cerebellum, and pyramidal tract degeneration was noted in the brain stem and spinal cord (Isshiki et al., 1994; Nakazato et al., 1991). Recently, we had the opportunity to learn of the third patient from this GSS105 family. This case was also subjected to autopsy limited to the brain, and a detailed neuropathological assessment was possible. The paraffin blocks of the previous two cases had been stored in whole in our institution (Saitama Medical University). Given such a situation, we decided to undertake a comprehensive study that aims to compare the clinicopathological profiles among the three cases.

GSS is known to be associated with hyperphosphorylated tau (phospho‐tau: p‐tau) deposition (Alzualde et al., 2010; Colucci et al., 2006; Ghetti et al., 1995, 1996, 1994; Hsiao et al., 1992; Ichimiya et al., 1994; Ikeda, Yanagisawa, Glenner, & Allsop, 1992; Ishizawa et al., 2002; Kitamoto, Lizuka, et al., 1993; Piccardo et al., 1998; Tranchant et al., 1997). Typically, p‐tau lesions are present in the form of neurofibrillary tangles (NFTs), dystrophic neurites (DNs), and/or neuropil threads (NTs) in or around PrP‐plaques. In a previous report of a GSS P102L (GSS102) patient complicated with dementia (Ishizawa et al., 2002), a full‐blown pathology comprised of PrP, p‐tau, and beta‐amyloid (Aβ) was present, and an interesting interaction of PrP, p‐tau, and Aβ was suggested. In a preliminary study of the present GSS105 cases, a considerable amount of p‐tau was found. A comprehensive analysis using single‐ and multiple‐labeling immunohistochemistry, which aims to further clarify the topological relationship among the three proteins, PrP, p‐tau, and Aβ, is another theme of this study.

## 2 MATERIALS AND METHODS

### 2.1 Case materials and clinical history

**Figure 1:**
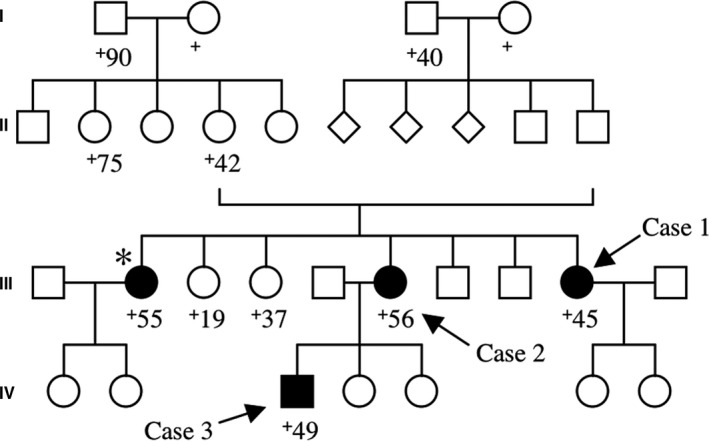
The family tree of the three cases is shown. Other than the three cases analyzed in this study (arrows), there is another sibling (*), a 55‐year‐old woman at death, who showed spastic paraparesis, dementia, and cerebellar dysfunction. Square, male; circle, female; diamond, an individual whose information of sex is unavailable; +, dead. Closed symbols indicate an individual with definite or possible GSS105‐associated symptoms

The family tree of the three kindred is shown in Figure 1. Case 1 and Case 2 are sisters, and Case 3 is the son of Case 2. The parents of Case 1 and Case 2 are a married couple between cousins. Although the details are unknown, there is another sibling of Case 1 and Case 2, who was affected with spastic paraparesis, dementia, and cerebellar dysfunction (Figure 1). The clinicopathological and genetic profiles of Case 1 (Isshiki et al., 1994; Kitamoto, Amano, et al., 1993; Nakazato et al., 1991) and Case 2 (Isshiki et al., 1994; Kitamoto, Amano, et al., 1993) were previously reported in Japanese (Isshiki et al., 1994; Nakazato et al., 1991) and English (Kitamoto, Amano, et al., 1993). The genetic analysis confirmed a diagnosis of GSS P105L for this family (Patient No. 1 and Patient No. 2 designated by Kitamoto, et al (Kitamoto, Amano, et al., 1993) correspond to Case 1 and Case 2 in this report, respectively). Case 3 is the virgin case that remains to be reported.

#### 2.1.1 Case 1

Since the clinical history of this patient, a 45‐year‐old woman at death, is already available elsewhere in Japanese (Nakazato et al., 1991), this report only describes the outline of it. At the age of 38, she manifested with difficulty in walking. A neurological examination 6 months after the onset disclosed spastic gait coupled with spasticity and pyramidal signs in both legs. A clinical diagnosis of “familial spastic paraparesis” was made. Two years after the onset, she became unable to walk. Soon dysarthria, emotional incontinence, and tremor of the tongue and fingers appeared. Eventually, she became severely demented and died of aspiration pneumonia 6 years after the onset.

#### 2.1.2 Case 2

Since the clinical history of this patient, a 56‐year‐old woman at death is already available elsewhere in Japanese (Isshiki et al., 1994), this report only describes the outline of it. When she was 5 years old, she was affected with poliomyelitis. Since then her right hand remained paralytic, but there was no problem in her daily life. At the age of 44, she manifested with difficulty in walking, and 4 months later, she became almost unable to walk. Soon dysarthria became evident. About 2 years after the onset, a clinical diagnosis of “familial spastic paraparesis” was made, and thereafter, she became bedridden. Then, she was admitted to a psychiatric hospital for the management of severe emotional disturbance. The pyramidal signs in the upper and lower limbs, chorea‐like movement in the left upper limb, apathy, and dementia followed, and she died at the age of 56, 12 years after the onset.

#### 2.1.3 Case 3

This patient, a 49‐year‐old man at death, is the son of Case 2. When he was 47 years old, he suffered from hemorrhoids and had them surgically removed. But after the operation, he repeatedly complained of anal pain and kept on consulting several hospitals. Soon, the family members found his way of walking somewhat clumsy. About 1 year later, he presented with tremor in his fingers. About 2 years after the onset, his stereotyped behavior and speech became more apparent; he walked around the same place at the same time each day; and he dropped in at the same store and bought the same foods (bread and cola). There was an episode where he complained of lucency of his teeth, and he consulted a dentist three days in a row. He began to make comments like “I have been deceived,” or “I have been robbed.” He became restless and easily agitated, which culminated in an attempted strangulation of his wife. He was hospitalized and remained conscious but did not utter any words. There was a tremor in the upper limbs, and there was myoclonus, which disappeared later, in the lower limbs. In all extremities, deep tendon reflexes were exaggerated but there was no paresis. There were no signs of sensory and cerebellar impairment. During the hospitalization, he relentlessly complained of anal pain and repeatedly ate the same foods (hamburgers and cola). The brain magnetic resonance imaging (MRI) showed mild frontotemporal atrophy, but did not disclose any signal abnormalities on diffusion MRI. The complaint of anal pain was so tenacious that oral morphine was introduced. But he unexpectedly passed away due to paralytic intestinal obstruction leading to septic shock. The whole clinical course was about 2 years (2 years and 3 months). The P105L point mutation of *PRNP* coupled with codon 129 polymorphism (Val/Met), which is identical to that of Case 1 and Case 2 (Kitamoto, Amano, et al., 1993), was detected by a genetic analysis using blood samples. An autopsy limited to the brain was performed.

#### 2.1.4 Tabulation of the clinical profiles compared among the three cases

Table 1 summarizes representative symptoms and some laboratory data of the three cases with the help of previous papers (Isshiki et al., 1994; Kitamoto, Amano, et al., 1993; Nakazato et al., 1991) and medical records available in our institution.

**Table 1:** Comparative clinical profiles among the three cases

	Case 1	Case 2	Case 3
Sex	F	F	M
Age at onset (years old)	38	44	47
Disease duration (years)	6	12	2
Age at death (years old)	45	56	49
Cause for death	Aspiration pneumonia	NA	Paralytic intestinal obstruction (Septic shock)
Initial presenting symptom	Spastic paraparesis	Spastic paraparesis	Stereotyped behavior and speech
Other subsequent symptoms	Dysarthria Emotional incontinence Tremor Dementia	Dysarthria Emotional disturbance Chorea‐like movement Apathy Dementia	Clumsy walking Tremor and Myoclonus Restlessness and Agitation
Myoclonus	(−)	(−)	(+, transiently)
Electroencephalogram	PSD (−)	PSD (−)	PSD (−)
Brain MRI	NA	NA	Frontotemporal atrophy Signal abnormality on diffusion MRI (−)
CSF (tau and 14‐3‐3 protein)	NA	NA	Tau and 14‐3‐3 protein: WNL
*PRNP*	Codon 129 polymorphism (Val/Met) P105L (on the Val^129^ allele)	Codon 129 polymorphism (Val/Met) P105L (on the Val^129^ allele)	Codon 129 polymorphism (Val/Met) P105L (on the Val^129^ allele)

Note

M, male; F, female; NA, not available; (+), present; (−), absent; PSD, periodic synchronous discharge; MRI, magnetic resonance imaging; CSF, cerebrospinal fluid; WNL, within normal limits; *PRNP*, prion protein gene.

### 2.2 Preparation of paraffin blocks and histological evaluation

The paraffin blocks of Case 1 and Case 2, which had been stored in Saitama Medical University, were retrieved for re‐evaluation of histology and immunohistochemistry; however, as PrP in these blocks had not been detoxicated before, the detoxication step by formic acid treatment was necessary. The paraffin blocks were immersed several times in xylene for deparaffinization then were immersed in ethanol, and finally in tap water. The deparaffinized blocks were immersed in 100% formic acid for 1 hr. After this disinfection step, the blocks were paraffinized again. With respect to Case 3, the brain was fixed in 10% buffered formalin, and representative sections were sliced. The sections were immersed in concentrated formic acid (98%) for 1 hr, washed in tap water, and embedded in paraffin.

The histology was evaluated using hematoxylin and eosin (HE), Klüver–Barrera (KB), HE‐Luxol fast blue (LFB), and/or Bodian stains. Gallyas silver stain was applied to all cases to evaluate neurofibrillary pathology.

### 2.3 Single‐ and multiple‐labeling immunohistochemistry for PrP, p‐tau, and Aβ

Primary antibodies used for single‐labeling immunohistochemistry were as follows: mouse monoclonal antibodies for PrP (3F4; 1:1,000 or 1:2,000; Dako, Carpinteria, CA, USA, (Kascsak et al., 1987)), p‐tau (AT8; 1:1,000; Innogenetics, Ghent, Belgium; Goedert, Jakes, & Vanmechelen, 1995; Mercken et al., 1992), three repeat tau (RD3; 1:1,000; Upstate Biotechnology, Lake Placid, NY, USA; de Silva et al., 2003), four repeat tau (RD4; 1:100; Upstate Biotechnology; de Silva et al., 2003), and Aβ (4G8; 1:20,000; Senetek, St. Louis, MO, USA; Kim et al., 1990). Antigen retrieval pretreatment was as follows: hydrolytic autoclaving in 1 mM HCl (121℃, 10 min) for 3F4, hydrolytic autoclaving in 10 mM EDTA (121℃, 10 min) for RD3 and RD4, and immersion in concentrated formic acid (98%, room temperature, 5 min) followed by washing in phosphate buffered saline (PBS) for 4G8. Single‐labeling immunohistochemistry was carried out as follows: the deparaffinized and rehydrated 5 μm‐thick sections were treated with the antigen retrieval pretreatment according to the primary antibody, washed in PBS, and incubated with the primary antibody (37℃, 60 min). Then, the sections were washed in PBS and treated with 3% hydrogen peroxide (room temperature, 10 min). After washing in PBS, the sections were incubated with a secondary antibody kit (Dako ChemMate EnVision kit/HRP (DAB)) (room temperature, 30 min). After washing in PBS, the sections were visualized with diaminobenzidine (DAB).

Double‐labeling immunohistochemistry was carried out with the following combination of primary antibodies (a mouse monoclonal antibody and a rabbit polyclonal antibody): AT8 and a rabbit polyclonal anti‐PrP antibody (PrP‐N; 1:2,000; gifted from Dr. Kitamoto; Kitamoto, Muramoto, Hilbich, Beyreuther, & Tateishi, 1991), 4G8 and PrP‐N, and/or a rabbit polyclonal anti‐tau antibody (1:1,000, A 024, Dako) and 4G8. PrP‐N is an excellent antibody, as is 3F4, which can be used to visualize PrP‐plaques (Kitamoto et al., 1991). The use of PrP‐N and 3F4 in immunohistochemistry is exchangeable, and both antibodies were successfully applied to single‐ and multiple‐labeling immunohistochemistry in a previous study of GSS (Ishizawa et al., 2002). First, the deparaffinized and rehydrated sections were incubated with one of the primary antibodies (37℃, 60 min), treated with a secondary antibody kit (Histofine Simple Stain AP (multi); Nichirei Biosciences, Tokyo, Japan) (room temperature, 30 min), and visualized with fast red (Fast Red II Substrate Kit; Nichirei Biosciences). Next, the sections were incubated with the other primary antibody (37℃, 60 min), treated with 3% hydrogen peroxide (room temperature, 10 min), incubated with another secondary antibody kit (Histofine Simple Stain MAX‐PO (R) or Histofine Simple Stain MAX‐PO (M)) (room temperature, 30 min), and visualized with DAB. Antigen retrieval pretreatment was applied before the incubation with the corresponding primary antibody.

Triple‐labeling immunohistochemistry was carried out with AT8 (p‐tau), 4G8 (Aβ), and PrP‐*N* (PrP) in Case 2, where numerous deposits of p‐tau, Aβ, and PrP were identified. First, the sections were incubated with AT8 (37℃, 60 min), then with a secondary antibody kit (Histofine Simple Stain AP (multi)) (room temperature, 30 min), and visualized with fast red. Second, after the treatment with 0.01 M citrate buffer (95℃, pH6, 10 min) for the dissolution of AT8, the sections were treated with the antigen retrieval pretreatment for 4G8 and then incubated with 4G8 (37℃, 60 min). After the treatment with 3% hydrogen peroxide (room temperature, 10 min), the sections were incubated with a secondary antibody kit (Histofine Simple Stain MAX‐PO (M)) (room temperature, 30 min) and visualized with DAB. Third, the sections were treated with the antigen retrieval pretreatment for PrP‐N and then incubated with PrP‐N (a rabbit polyclonal antibody). After the treatment with 3% hydrogen peroxide (room temperature, 10 min), the sections were incubated with a secondary antibody (biotinylated anti‐rabbit IgG and peroxidase‐conjugated streptavidin, Code 426012 and 426062; Nichirei Biosciences), which was then followed by visualization with a peroxidase substrate (HistoGreen; E109, Cosmo Bio, Tokyo, Japan).

### 2.4 Comparative semi‐quantitative analyses of tissue injuries and 3F4‐immunoreactive PrP‐plaques among the three cases

To compare the three cases, the neuroanatomic structures, including the cerebral cortices and white matter, basal ganglia (putamen, globus pallidus, and/or caudate nucleus), thalamus, hippocampus, brain stem, cerebellar cortices and white matter, and spinal cord, were semi‐quantified on HE, KB, and HE‐LFB sections for tissue injuries (neuronal loss, gliosis, spongiform change, and/or myelin loss/pallor) in the most affected portion as ± (none or few: minimal), + (mild), ++ (moderate), and +++ (severe). As for PrP‐plaques visualized by 3F4‐immunohistochemistry, they were semi‐quantified in the portion showing the highest density of immunoproducts as ‐ (none), + (sparse), ++ (moderate), and +++ (frequent) with a reference to the semi‐quantification strategy for senile plaques in Alzheimer’s disease (AD) (CERAD) (Fillenbaum et al., 2008; Mirra et al., 1991). In the cerebral cortices, as the PrP‐plaques were distributed in a layer‐dependent manner, the semi‐quantification was subdivided into the superficial (layers I and II), middle (III and IV), and deep (V and VI) cortical layers.

## 3 RESULTS

### 3.1 Histology and single‐labeling immunohistochemistry for PrP

#### 3.1.1 Case 1

**Figure 2:**
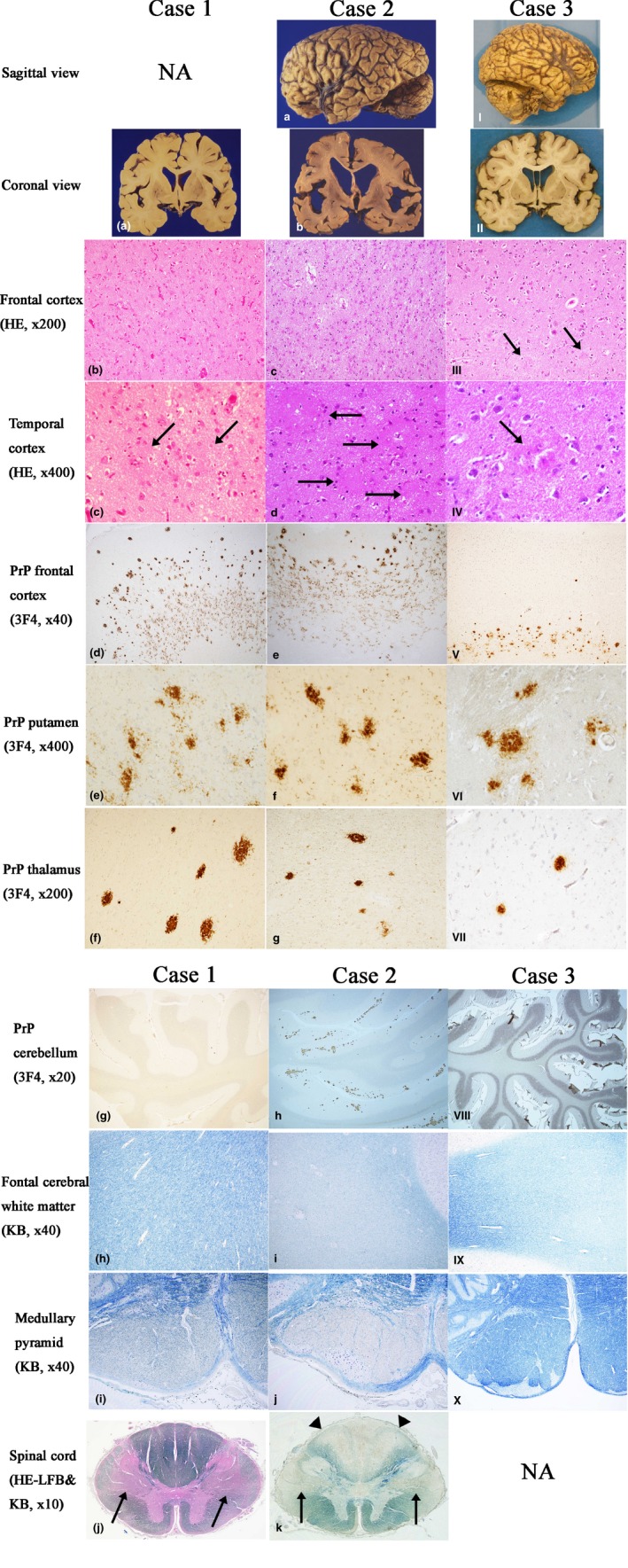
The comparative macroscopic and microscopic findings among the three cases. Left column (Case 1, a–j), middle column (Case 2, a–k), right column (Case 3, I–X). The 1st and 2nd rows show macroscopic findings of each case. The 3rd to 11th rows show microscopic findings of each case. NA, not available. Case 1: (a) The brain is mildly atrophic in the frontal and temporal lobes. (b) In the cerebral cortices, neuronal loss and gliosis are noted (HE). (c) PrP‐plaques in the temporal cortex (arrows, HE). (d) PrP‐plaques in the frontal cortex, particularly along the deep cortical layers (3F4‐immunostain). (e) PrP‐plaques in the putamen. (3F4‐immunostain). (f) PrP‐plaques in the thalamus (3F4‐immunostain). (g) In the cerebellum, PrP‐plaques are absent. (3F4‐immunostain). (h) In the cerebral white matter, myelin is well‐preserved (KB). (i) The medullary pyramid shows myelin loss/pallor (KB). (j) The spinal cord shows myelin loss/pallor in the pyramidal tract (arrows, HE‐LFB). Case 2: (a) The brain is severely atrophic, especially in the frontal and temporal lobes. (b) On coronal sections, the cortical ribbon is thin, the cerebral white matter and corpus callosum are atrophic and discolored, and the lateral ventricle is dilated. (c) In the cerebral cortices, neuronal loss and gliosis are severe. A mild degree of spongiform change is also noted. (HE). (d) PrP‐plaques in the temporal cortex. (arrows, HE). (e) PrP‐plaques in the frontal cortex, particularly along the deep cortical layers (3F4‐immunostain). (f) PrP‐plaques in the putamen (3F4‐immunostain). (g) PrP‐plaques in the thalamus (3F4‐immunostain). (h) In the cerebellum, a number of PrP‐plaques are present (3F4‐immunostain). (i) The cerebral white matter shows myelin loss/pallor (KB). (j) The medullary pyramid shows myelin loss/pallor (KB). (k) The spinal cord shows marked myelin loss/pallor in the pyramidal tract (arrows) and the posterior column (arrowheads) (KB). Case 3: (I) The brain is mildly atrophic in the frontal and temporal lobes. (II) On coronal sections, the gyri are mildly wide and the lateral ventricle is mildly dilated. (III) The tissue injuries in the cerebral cortices, including neuronal loss and gliosis, are minimal except for the presence of PrP‐plaques (arrows) (HE). (IV) A PrP‐plaque in the temporal cortex (arrow, HE). (V) PrP‐plaques in the frontal cortex, particularly along the deep cortical layers (3F4‐immunostain). (VI) PrP‐plaques in the putamen (3F4‐immunostain). (VII) PrP‐plaques in the thalamus (3F4‐immunostain). (VIII) In the cerebellum, PrP‐plaques are absent (3F4‐immunostain). (IX) The cerebral white matter shows mild myelin loss/pallor (KB). (X) In the medullary pyramid, myelin is well‐preserved (KB)

The brain weighed 1,060 g. Grossly, the brain was mildly atrophic in the frontal and temporal lobes (Figure 2a). In the cerebral cortices, neuronal loss and gliosis were present (Figure 2b), particularly along the deep cortical layers, but spongiform change was minimal. A number of PrP‐plaques were noted (Figure 2c,d). While the PrP‐plaques in the superficial or middle cortical layers were compact and well‐demarcated, those in the deep cortical layers were blurry, ill‐defined, and confluent (Figure 2d). In the basal ganglia, hippocampus, and thalamus, tissue injuries were minimal, but a considerable number of PrP‐plaques were visualized by immunostaining for PrP (Figure 2e,f). In the cerebellum, tissue injuries were minimal and there was no deposition of PrP‐plaques (Figure 2g). The cerebral white matter was well‐preserved (Figure 2h). In the brain stem and spinal cord, the pyramidal tract showed severe myelin loss/pallor (Figure 2i,j).

#### 3.1.2 Case 2

The brain, whose weight was 975 g, was considerably atrophic, particularly in the frontal and temporal lobes (Figure 2a). The cortical ribbon was thin, the cerebral white matter, including the corpus callosum, was atrophic and discolored, and the lateral ventricle was markedly dilated (Figure 2b). Neuronal loss and gliosis were severe, particularly along the deep cortical layers, and a mild degree of spongiform change was noted (Figure 2c). A number of PrP‐plaques were present (Figure 2d,e). As in Case 1, while the PrP‐plaques in the superficial or middle cortical layers were relatively well‐defined and compact, those in the deep cortical layers were ill‐defined, amorphous, and confluent (Figure 2e). The basal ganglia, hippocampus, and thalamus showed a mild to moderate degree of neuronal loss and gliosis, where a number of PrP‐plaques were present (Figure 2f,g). In the cerebellum, while tissue injuries were not evident on histological sections, a number of PrP‐plaques were visualized by PrP‐immunohistochemistry (Figure 2h). The cerebral white matter showed severe myelin loss/pallor with relative preservation of U‐fibers (Figure 2i). In the brain stem, the pyramidal tract showed severe myelin loss/pallor (Figure 2j). The spinal cord showed severe myelin loss/pallor in the pyramidal tract as well as in the posterior column (Figure 2k).

**Figure 3:**
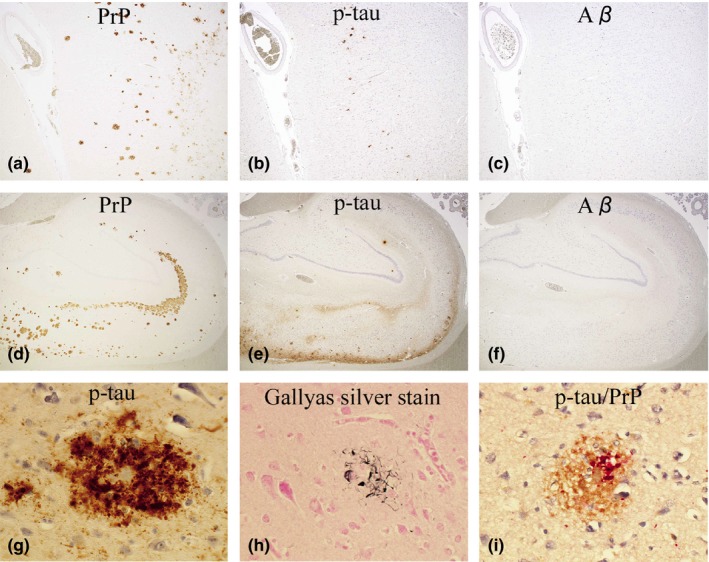
Case 1: (a–c, d–f) The distribution of PrP (a, d), p‐tau (b, e), and Aβ (c, f) in the temporal cortex (a–c) and hippocampus (d–f) is shown. The photos are taken from an identical area for a–c and d–f. P‐tau (b&e, AT8‐immunostain) seems considerably overlapped with PrP (a&d, 3F4‐immunostain), whereas Aβ (c&f, 4G8‐immunostain) is completely absent (Original magnification: a–c, ×40; d–f, ×20). (g) P‐tau‐positive dystrophic neurites (DNs) are aggregated around a PrP‐plaque (AT8‐immunostain, temporal cortex, ×600). (h) A fraction of DNs are argyrophilic (Gallyas silver stain, temporal cortex, ×600). (i) By double‐immunostaining with AT8 and PrP‐N, the Colocalization of p‐tau with PrP‐plaques is confirmed (p‐tau, red; PrP, brown. Occipital cortex, ×600)

#### 3.1.3 Case 3

The brain, which weighed 1,560 g, was mildly atrophic in the frontal and temporal lobes (Figure 2I and II). In the cerebral cortices, neuronal loss and gliosis as well as spongiform change were only minimal (Figure 2III). PrP‐plaques were identified, particularly along the deep cortical layers (Figure 2III–V). In the basal ganglia, hippocampus, and thalamus, a variable number of PrP‐plaques were visualized by PrP‐immunohistochemistry (Figure 2VI,VII). The cerebellum was unremarkable with no deposition of PrP‐plaques (Figure 2VIII). The cerebral white matter showed mild myelin loss/pallor (Figure 2IX). The brain stem, including the pyramidal tract, was unremarkable (Figure 2X).

### 3.2 Comparative semi‐quantitative analyses of tissue injuries and PrP‐plaques visualized by 3F4‐immunohistochemistry

The tissue injuries and PrP‐plaques visualized by 3F4‐immunohistochemistry, which were semi‐quantified for the three cases, are presented in Tables 2 and 3, respectively. The tissue injuries were the most prominent in Case 2, which was followed by Case 1 and then by Case 3. The pyramidal tract in Case 1 and Case 2 was severely affected, while that in Case 3 remained intact. Similarly, the PrP‐plaques visualized by 3F4‐immunohistochemistry were the most numerous in Case 2, which was followed by Case 1 and then by Case 3. Particularly in Case 2, a large number of PrP‐plaques were noted not only in the cerebrum but also in the cerebellum. Even in Case 3, who was the youngest and had the shortest clinical course, there were a fair number of PrP‐plaques in the cerebrum, especially along the deep cortical layers.

**Table 2:** Comparative semi‐quantitative analysis of tissue injuries among the three cases

	**Case 1** **(45 yo, F)**	**Case 2** **(56 yo, F)**	**Case 3** **(49 yo, M)**
Brain weight (g)	1,060 g	975 g	1,560 g
Disease duration (years)	6	12	2
Cerebral cortex			
Neuronal loss/Gliosis	++	+++	±
Spongiform change	±	+	±
Cerebral white matter			
Myelin loss/pallor	±	+++	+
Basal ganglia			
Neuronal loss/Gliosis	±	+	±
Spongiform change	±	+	±
Hippocampus			
Neuronal loss/Gliosis	±	+	±
Spongiform change	±	±	±
Thalamus			
Neuronal loss/Gliosis	±	++	±
Spongiform change	±	+	±
Brain stem			
Myelin loss/pallor (pyramidal tract)	+++	+++	±
Cerebellar cortex			
Neuronal loss/Gliosis	±	±	±
Spongiform change	±	±	±
Cerebellar white matter			
Myelin loss/pallor	±	±	+
Spinal cord			
Myelin loss/pallor (pyramidal tract)	+++	+++	NA
Myelin loss/pallor (posterior column)	±	+++	NA

Note

yo, years old; M, male; F, female; NA, not available; ±, none or few (minimal); +, mild; ++, moderate; +++, severe.

**Table 3:** Comparative semi‐quantitative analysis of PrP‐plaques visualized by 3F4‐immunohistochemistry among the three cases

	Case 1 (45 yo, F)	Case 2 (56 yo, F)	Case 3 (49 yo, M)
Brain weight (g)	1,060 g	975 g	1,560 g
Disease duration (years)	6	12	2
Cerebral cortex			
Frontal			
S	+	++	‐
M	++	+++	+
D	+++	+++	+++
Temporal			
S	+	++	‐
M	++	+++	+
D	+++	+++	++
Parietal			
S	+	NA	+
M	++	NA	++
D	+++	NA	+++
Occipital			
S	+	+	+
M	++	++	++
D	+++	+++	+++
Basal ganglia	+++	+++	+++
Hippocampus	+++	+++	‐
Thalamus	++	++	+
Cerebellar cortex	‐	+++	–

Note

yo, years old; M, male; F, female; NA, not available; S, superficial; M, middle; D, deep; –, none; +, sparse; ++, moderate; +++, frequent.

### 3.3 Single‐ and multiple‐labeling immunohistochemistry for PrP, p‐tau, and Aβ

**Figure 4:**
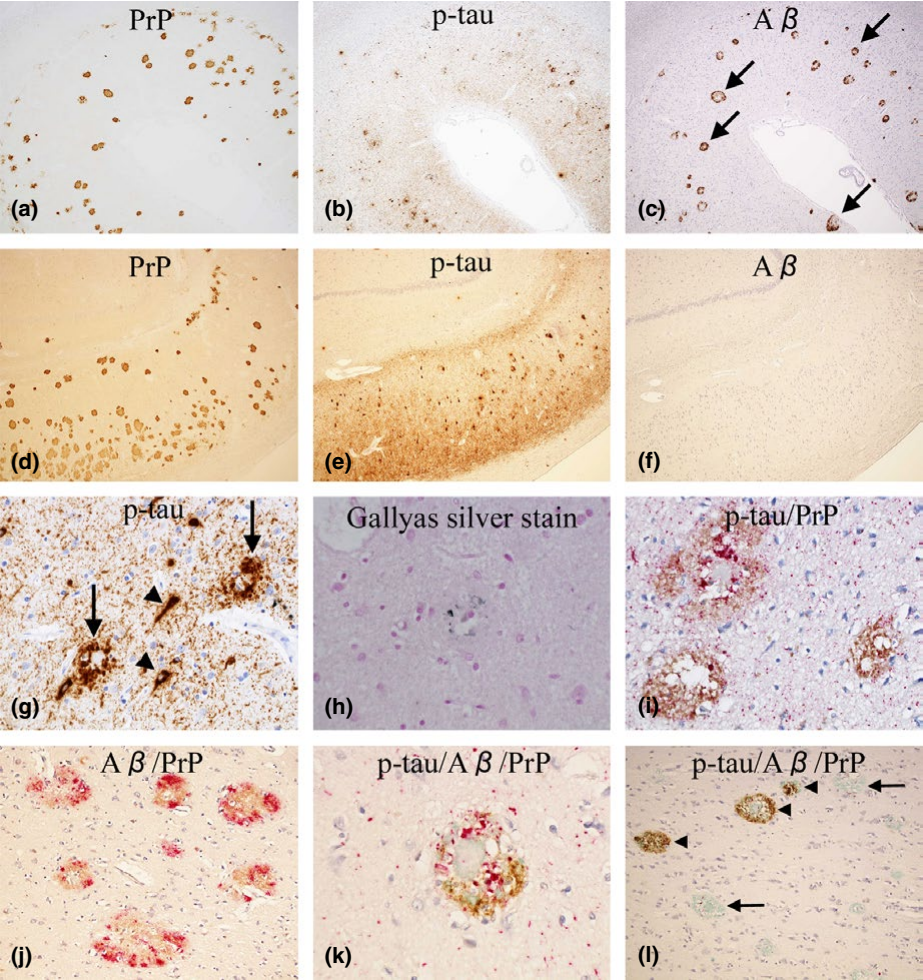
Case 2: (a–c, d–f) The distribution of PrP (a, d), p‐tau (b, e), and Aβ (c, f) in the temporal cortex (a–c) and hippocampus (CA1 to subiculum, d–f) is shown. The photos are taken from an identical area for a–c and d–f. In the temporal cortex (a–c), p‐tau (b, AT8‐immunostain) and Aβ (c, 4G8‐immunostain) seem considerably overlapped with PrP (a, 3F4‐immunostain). Notably, most deposits of Aβ have an immuno‐negative central core (arrows, c). In the hippocampus (d–f), p‐tau (e, AT8‐immunostain) seems considerably overlapped with PrP (d, 3F4‐immunostai), whereas Aβ (f, 4G8‐immunostain) is totally absent. (Original magnification: a–c, ×40; d–f, ×40). (g) P‐tau‐positive dystrophic neurites (DNs) around PrP‐plaques (arrows), neurofibrillary tangles (arrowheads), and neuropil threads are commonly seen (AT8‐immunostain, temporal cortex, ×400). (h) A small fraction of DNs around PrP‐plaques are argyrophilic (Gallyas silver stain, frontal cortex, x600). (i) By double‐immunostaining with AT8 and PrP‐N, the colocalization of p‐tau with PrP‐plaques is confirmed. (p‐tau, red; PrP, brown. Temporal cortex, x400). (j) By double‐immunostaining with 4G8 and PrP‐N, the deposition of Aβ around PrP‐plaques is confirmed. Note that most Aβ is colocalized with PrP. (Aβ, red; PrP, brown. Temporal cortex, ×200). (k) By triple‐immunostaining with AT8, 4G8, and PrP‐N, the colocalization of p‐tau, Aβ, and PrP is confirmed (p‐tau, red; Aβ, brown; PrP, green. Temporal cortex, ×600). (l) By triple‐immunostaining with AT8, 4G8 and PrP‐N, it is also shown that PrP, with or without p‐tau, can be present without Aβ (arrows); on the other hand, Aβ, with or without p‐tau, cannot be present without PrP (arrowheads), suggesting that PrP deposition is likely a precursor event to Aβ deposition. (p‐tau, red; Aβ, brown; PrP, green. Temporal cortex, ×600)

**Figure 5:**
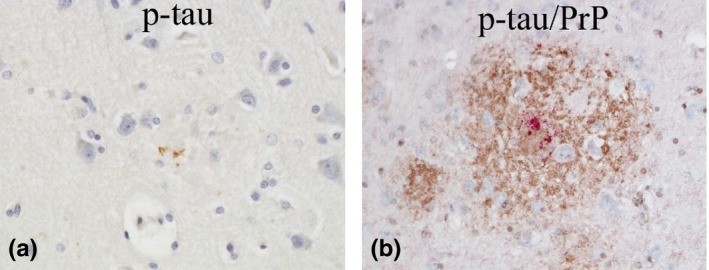
Case 3: (a) Although minimal, p‐tau associated with PrP‐plaques is identified. (AT8‐immunostain, frontal cortex, original magnification ×600). (b) By double‐immunostaining with AT8 and PrP‐N, the colocalization of p‐tau with PrP‐plaques is confirmed (p‐tau, red; PrP, brown. Temporal cortex, ×400)

The frontal lobe, temporal lobe, parietal lobe (except for Case 2), occipital lobe, hippocampus, and/or cerebellum were analyzed for the topological relationship of PrP, p‐tau, and Aβ (Figures 3, 4, 5).

In Case 1 (Figure 3), PrP‐plaques and p‐tau were scattered in the cerebral cortices (Figure 3a,b) and hippocampus (Figure 3d,e). P‐tau was by far the most numerous in the temporal lobe and hippocampus, but was absent in the cerebellum. Aβ was totally absent in all the areas studied (Figure 3c,f). Interestingly, the distribution of PrP‐plaques and p‐tau seemed considerably overlapped (Figure 3a,b and d,e). At high magnification, p‐tau comprised of NFTs, DNs, and/or NTs, seemed associated with PrP‐plaques (Figure 3g). A fraction of p‐tau around PrP‐plaques was argyrophilic (Figure 3h). By double‐immunostaining, the colocalization of p‐tau with PrP‐plaques was confirmed (Figure 3i).

In Case 2 (Figure 4), similarly to Case 1, PrP‐plaques and p‐tau were noted in the cerebral cortices (Figure 4a,b) and hippocampus (Figure 4d,e), but the amount of p‐tau was much more than in Case 1. Similarly to Case 1, p‐tau was by far the most numerous in the temporal lobe and hippocampus, but was absent in the cerebellum. In this case, the deposition of Aβ was also noted in the cerebral cortices (Figure 4c), but not in the hippocampus (Figure 4f) and cerebellum. Notably, the majority of Aβ had an immuno‐negative central core (Figure 4c), which later turned out to be PrP (Figure 4j). Similarly to Case 1, the distribution of PrP‐plaques and p‐tau seemed considerably overlapped (Figure 4a,b and d,e). P‐tau comprised of NFTs, DNs, and/or NTs seemed associated with PrP‐plaques (Figure 4g), and a small fraction of it was argyrophilic (Figure 4h). By double‐immunostaining, the colocalization of p‐tau with PrP‐plaques was confirmed (Figure 4i). The deposits of Aβ, if present at all, were mostly colocalized with PrP‐plaques (Figure 4j). By triple‐immunostaining, the colocalization of p‐tau, Aβ, and PrP was confirmed (Figure 4k). While PrP, with or without p‐tau, could be present without Aβ, Aβ, with or without p‐tau, could not be present without PrP (Figure 4l), suggesting that PrP deposition is likely a precursor event to Aβ deposition.

In Case 3 (Figure 5), a minimal amount of p‐tau was noted in the cerebral cortices (Figure 5a), all of which, by double‐labeling immunohistochemistry, was colocalized with PrP‐plaques (Figure 5b). Aβ was totally absent in all the areas studied.

### 3.4 Single‐labeling immunohistochemistry for three repeat tau (RD3) and four repeat tau (RD4)

**Figure 6:**
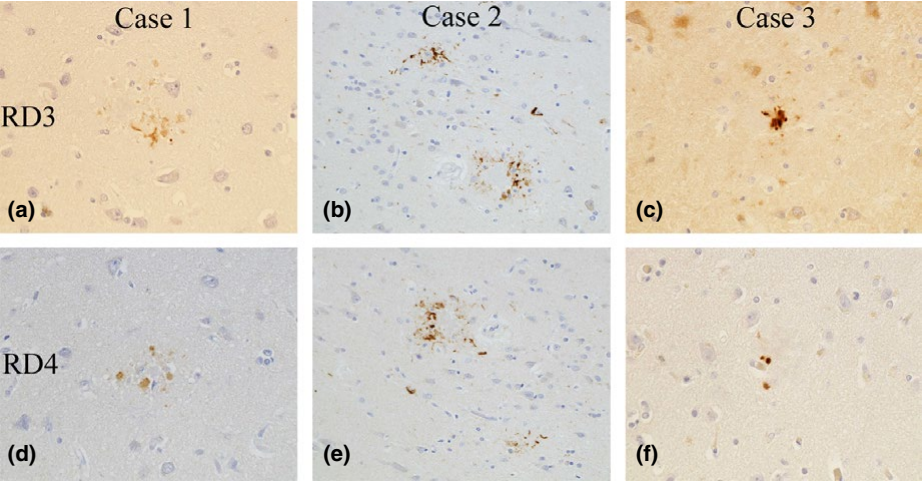
In all cases, both three repeat tau (RD3, a–c) and four repeat tau (RD4, d–f) are associated with PrP–plaques. (RD3‐ & RD4‐immunostain, original magnification: a&d, c&f ×600; b&e ×400)

In all cases, both three repeat tau (RD3) and four repeat tau (RD4) were associated with PrP‐plaques (Case 1, Figure 6a,d; Case 2, Figure 6b,e; Case 3, Figure 6c,f).

## 4 DISCUSSION

Clinically, Case 1 and Case 2, who were female siblings, manifested with spastic paraparesis, which was then followed by dysarthria, psychiatric symptoms, and dementia. Case 3, who was the son of Case 2, manifested with psychiatric symptoms, such as stereotyped speech and behavior; but in contrast to Case 1 and Case 2, there were no signs of spastic paraparesis during the whole clinical course.

Although the clinical picture of GSS105 is usually predominated by gait disturbance (spastic paraparesis), psychiatric symptoms, and dementia (Amano et al., 1992; Isshiki et al., 1994; Itoh et al., 1994; Kitamoto, Amano, et al., 1993; Kubo et al., 1995; Nakazato et al., 1991; Yamada et al., 1993), there is a substantial variation to it even within the same family (Iwasaki, Kizawa, Hori, Kitamoto, & Sobue, 2009; Koshi Mano et al., 2016; Shiraishi, Mizusawa, & Yamada, 2002; Yamada et al., 1999, 1995; Yamazaki et al., 1999). In a GSS105 family reported elsewhere (Shiraishi et al., 2002; Yamazaki et al., 1999), one member presented with gait disturbance (gait apraxia) that was followed by mutism (Yamazaki et al., 1999), and the other presented with sensory and psychiatric symptoms, including a persistent complaint of pains in various parts of the body, which were then followed by memory disturbance, delusion, and gait disturbance (Shiraishi et al., 2002). A recent paper described three GSS105 families (Families 1–3) whose clinical profile included atypical parkinsonism (Koshi Mano et al., 2016), a rare symptom of GSS105. In each family, the affected individuals had different symptoms. For example, in Family 1, three of the four affected individuals manifested with gait disturbance, and the remaining one manifested with dysesthesia and gait disturbance. Not only parkinsonism but also other neurological signs, such as spasticity, ataxia, involuntary movement, dementia, or emotional instability, were variably noted in all families. Taken together with these reports and others (Amano et al., 1992; Iwasaki et al., 2009; Kubo et al., 1995; Yamada et al., 1999, 1995), the present study asserts a wide spectrum of symptoms associated with GSS105 even within the same family. The same is true for other mutations of GSS (Giovagnoli et al., 2008; Hsiao et al., 1991; Kovacs et al., 2001; Majtenyi, Brown, Cervenakova, Goldfarb, & Tateishi, 2000; Mastrianni et al., 1995; Nochlin et al., 1989; Piccardo et al., 1998; Popova et al., 2012; Webb et al., 2008). In this respect, though one point merits mention about the clinical profile of the present Case 3: this patient died of paralytic intestinal obstruction leading to septic shock about 2 years after the onset. Based on his clinical history, this comorbidity is more than likely the result, not of GSS105 itself, but of the oral administration of morphine. In fact, intestinal symptoms have rarely been reported in GSS105 (Iwasaki et al., 2009; Koshi Mano et al., 2016). The death of the patient is rather unexpected, and his clinical course, about 2 years in all, is much shorter than those expected for GSS105 (Iwasaki et al., 2009). This could have contributed to the lack of some clinical features compared to the other two cases. For example, although spastic paraparesis was not evident in this patient, it was recorded that his way of walking was clumsy and deep tendon reflexes were exaggerated. These neurological signs could have been a prelude to spastic paraparesis that could have been possible later if the patient had not prematurely passed away.

Pathologically, in Case 1 and Case 2, the cerebral cortices and pyramidal tract were severely affected. In contrast, in Case 3, the cerebral cortices were relatively preserved, and the pyramidal tract remained uninvolved.

The clinicopathological study of GSS105 with a focus of comparison between individuals within the same family is so far available in two families; one is no other than the present family, the two members of which were previously reported (Isshiki et al., 1994; Kitamoto, Amano, et al., 1993; Nakazato et al., 1991), and the other is the one reported by (Yamada et al. 1999; Itoh et al., 1994). The latter family is comprised of two siblings affected by the disease. One was a male patient whose initial symptom was clumsiness of the right hand at the age of 42, which was followed by spastic paraparesis. Subsequently, he showed signs of ataxia of the extremities, memory impairment, dysarthria, and apraxia. He died at the age of 53. At autopsy, the brain weighed 1,150 g and showed frontal atrophy. In the cerebral cortices, various sizes of compact and amorphous PrP‐plaques were noted, and the pyramidal tract was degenerated from the brain stem to the spinal cord. The other was the sister of the first patient, who initially presented with difficulty in writing because of tremulous movements of the upper extremities, which was followed by gait disturbance, involuntary movement of the legs, speech disturbance, and character changes. When she was 56 years old, she showed emotional and intellectual disturbance. Her speech was scanning with a small voice, and action myoclonus was prominent in the extremities. There was hyperreflexia of the legs. She died at the age of 58. At autopsy, the brain weighed 1,200 g. The histopathology of this patient was similar to that of her brother; in the cerebral cortices, various sizes of compact and amorphous PrP‐plaques were noted, and the pyramidal tract was degenerated from the brain stem to the spinal cord. Taken together with these reports, the present study indicates that the histopathology of GSS105 can considerably differ from individual to individual within the same family; and yet, it is a good reflection of the symptoms of each patient. Albeit with a few exceptions showing discordant clinicopathological correlations (Webb et al., 2008), the same is true for other mutations of GSS (Colucci et al., 2006; Ghetti et al., 1989; Majtenyi et al., 2000; Nochlin et al., 1989; Popova et al., 2012). In this respect, though, similarly to the first paragraph of this discussion, one point merits mention about the histological aspects of the present Case 3: this patient prematurely passed away about 2 years after the onset. Thus, for example, although the histological evidence of pyramidal tract involvement, as well as clinical evidence of spastic paraparesis, was not present, the histology corresponding to pyramidal tract involvement, as well as spastic paraparesis, could have been possible later if the patient had not prematurely passed away.

We studied the topological relationship of PrP, p‐tau, and Aβ in the three cases. In all cases, p‐tau lesions, that is, NFTs, DNs, and/or NTs were identified in or around PrP‐plaques. Particularly in Case 2, who was the oldest (56 years old) and had the longest clinical course (12 years), numerous p‐tau lesions were associated with PrP‐plaques, particularly in the temporal cortices and hippocampus. Furthermore, a large amount of Aβ, which was totally absent in Case 1 and Case 3, was almost invariably colocalized with PrP‐plaques. By triple‐labeling immunohistochemistry, the colocalization of PrP, p‐tau, and Aβ was confirmed.

GSS is a known condition where p‐tau is associated with PrP‐plaques. In particular, GSS145 (Y145Stop), 198 (F198S), and 217 (Q217R) are commonly associated with p‐tau deposition (Ghetti et al., 1995, 1996, 1994; Hsiao et al., 1992; Ichimiya et al., 1994; Ikeda et al., 1992; Kitamoto, Lizuka, et al., 1993). Although less common, GSS102, 105, 117 (A117V), 187 (H187R), 202 (D202N), and 218 (Y218N) are also relevant examples (Alzualde et al., 2010; Colucci et al., 2006; Ghetti et al., 1995; Ishizawa et al., 2002; Piccardo et al., 1998; Tranchant et al., 1997).

Previously, a full‐blown pathology comprised of PrP, p‐tau, and Aβ was reported in a GSS102 patient, a 44‐year‐old man with a 7‐year history of dementia (Ishizawa et al., 2002). Numerous p‐tau lesions, including NFTs, DNs, and NTs, were found in or around PrP‐plaques. An interesting feature of this case was that although a considerable amount of Aβ was found, the majority of it was not colocalized with PrP‐plaques. A computer‐assisted image analysis targeting the cerebral cortices disclosed a positive and significant correlation between PrP and p‐tau, but not between PrP and Aβ. A similar observation, where PrP was associated with p‐tau, but not with Aβ, was also reported in a fraction of GSS patients (Amano et al., 1992; Colucci et al., 2006; Ichimiya et al., 1994; Itoh et al., 1994; Kitamoto, Lizuka, et al., 1993; Yamada et al., 1999). In an in vitro experiment, the molecular interaction between tau and PrP was shown; the *N*‐terminus and repeat region of tau are actively involved in its interaction with PrP, and more specifically, the GSS‐related mutant of PrP, PrP102, was shown to have a higher tau‐binding activity than wild‐type PrP (Wang et al., 2008). It is plausible that PrP in GSS possesses an intrinsic ability to induce p‐tau deposition without the help of Aβ (Ishizawa et al., 2002; Reiniger et al., 2011). Concerning biochemical properties of tau in GSS, the paired helical filaments, which are morphologically identical to those of AD, are reported in GSS145, 198, and 217 (Ghetti et al., 1995, 1996, 1994 ). Antigenic profiles of NFTs in GSS145 and 198 are shown to be similar, if not identical, to those in AD by immnocytochemistry and immunoblot analysis (Ghetti et al., 1995, 1996, 1994 ; Giaccone et al., 1990; Tagliavini et al., 1993). In fact, the present study showed that p‐tau lesions associated with PrP‐plaques contained both three repeat tau and four repeat tau, just as in AD (Siddiqua & Margittai, 2010). Supposing PrP deposition were the primary pathology of GSS, p‐tau deposition in GSS could be regarded as a “secondary degeneration” due to PrP deposition, just as p‐tau deposition is likely a “secondary degeneration” due to Aβ deposition in AD (Hardy, Duff, Hardy, Perez‐Tur, & Hutton, 1998).

In this context, the finding of Case 2 merits particular attention. In this patient, a 56‐year‐old woman with a 12‐year history of the disease, the majority of Aβ was colocalized with PrP‐plaques, which was in sharp contrast to the GSS102 patient discussed above (Ishizawa et al., 2002). Aβ deposition associated with PrP‐plaques is also a known phenomenon in GSS (Ghetti et al., 1995, 1994; Ikeda et al., 1992; Miyazono, Kitamoto, Iwaki, & Tateishi, 1992; Tranchant et al., 1997); in GSS105, 117, 187, 198, 217, and 218, the patients can show colocalization of PrP and Aβ within the same plaques (Alzualde et al., 2010; Amano et al., 1992; Colucci et al., 2006; Ghetti et al., 1995, 1994; Ikeda et al., 1992; Itoh et al., 1994; Nochlin et al., 1989; Tranchant et al., 1997; Yamada et al., 1999; Yamazaki et al., 1999). There are experimental models showing that PrP can bind Aβ with high affinity (Lauren, Gimbel, Nygaard, Gilbert, & Strittmatter, 2009; Li, 2016). This phenomenon, however, seems dependent on the type of mutation of *PRNP*; in GSS145, PrP deposits are reported to be negative for Aβ (Ghetti et al., 1996; Ichimiya et al., 1994; Kitamoto, Lizuka, et al., 1993). Furthermore, it should be borne in mind that the deposition of Aβ depends, to a certain extent, on the individual and old age (Bugiani et al., 1993; Colucci et al., 2006; Miyazono et al., 1992; Ohgami, Kitamoto, Weidmann, Beyreuther, & Tateishi, 1991), since not all members of GSS117, 198 or 217 family show Aβ deposition (Bugiani et al., 1993; Colucci et al., 2006; Ghetti et al., 1995; Tranchant et al., 1997). In accordance with a previous report showing a high concurrence of prion disease pathology and AD pathology in Creutzfeldt‐Jacob‐disease (Tousseyn et al., 2015), the histopathology of the present Case 2 possesses strong evidence that Aβ deposition, as well as p‐tau deposition, could be directly or indirectly induced by PrP itself (“secondary degeneration”). Probably Aβ deposition within PrP‐plaques could facilitate p‐tau deposition further. This kind of patho‐mechanism should underlie the kaleidoscopic symptoms of GSS, which are always evolving and overlying one another.

## CONFLICT OF INTEREST

This study has no conflict of interest.
